# Saturation effects of the relationship between physical exercise and systemic immune inflammation index in the short-sleep population: a cross-sectional study

**DOI:** 10.1186/s12889-024-19432-7

**Published:** 2024-07-17

**Authors:** Yanwei You, Alimjan Ablitip, Yuquan Chen, Hao Ding, Keshuo Chen, Yicong Cui, Xindong Ma

**Affiliations:** 1 Division of Sports Science & Physical Education, Tsinghua University, Beijing, China; 2 School of Social Sciences, Tsinghua University, Beijing, China; 3 School of Public Health and Preventive Medicine, Faculty of Medicine, Nursing & Health Sciences, Monash University, Melbourne, VIC Australia; 4 IDG/McGovern Institute for Brain Research, Tsinghua University, Beijing, China

**Keywords:** Physical exercise, Systemic immune inflammation index, Short sleep, Cross-sectional study, Saturation effects

## Abstract

**Background:**

Short sleep can lead to an increase in inflammation and regular exercise has been shown to have a mitigation effect. However, the association between physical exercise (PE) and inflammation in the short sleep population is an unknown and intriguing issue.

**Methods:**

NHANES dataset spanning the years 2007 to 2018 were analyzed. To investigate the relationship mentioned above, we carried out multivariate linear regression models controlling for sociodemographic and lifestyles factors. The systemic immune inflammation index (SII) served as a reflection of inflammatory potential, calculated as the product of platelet count, neutrophil count, and divided by the lymphocyte count. Self-reported questionnaires were used to collect sleep and exercise information.

**Results:**

A total of 14,664 participants were included for final analysis. Across the three models, PE showed significant negative associations with SII as a continuous variable [Crude Model, β (95% CI): -1.261(-1.600, -0.922), *p* < 0.001; Model 1, β (95% CI): -1.005(-1.344, -0.666), *p* < 0.001; Model 2, β (95% CI): -0.470(-0.827, -0.112), *p* = 0.011]. The consistent nature of the findings persisted when investigating physical exercise (PE) as a categorized variable. By two-piecewise linear regression model, we calculated a saturation effect of PE with the inflection point as 2400 MET-minutes/week.

**Conclusion:**

This study suggested that performing no more than 2400 MET-minutes/week of PE was associated with lower SII levels in the short sleep population, while more PE might not bring additional benefits.

**Supplementary Information:**

The online version contains supplementary material available at 10.1186/s12889-024-19432-7.

## Introduction

In today’s fast-paced society, characterized by demanding lifestyles and work schedules, short sleep has emerged as a prevalent concern deserving heightened attention, particularly due to its association with mortality [1, 2]. Typically, no more than seven hours per night is defined as short sleep [3], which has been considered a risk factor for many chronic diseases, including diabetes [4], obesity [5], hypertension [6], and hypertriglyceridemia [7]. Sleep, a naturally recurring physiological process orchestrated by the central nervous system, has a rich history of supporting recovery from various diseases and infections, underscoring its pivotal role in immune system regulation. Emerging research reveals that short sleep duration disrupts the secretion patterns of both systemic and cellular inflammatory markers. For instance, it is associated with elevated levels of systemic interleukin-6 (IL-6) [8] and C-reactive protein (CRP) [9].

Physical exercise has been proven to improve overall health and reduces all-cause mortality risk [10]. Exercise can improve both physical and mental health, including cardiovascular fitness, circulation system, sleep quality, and negative mood [11–13]. For potential mechanism, previous studies have convincingly suggested regular exercise can induce a cell-autonomous anti-inflammatory response and modulate several signaling pathways, thereby reducing serum pro-inflammatory markers such as CRP [14], IFN-γ [15], IL-1β [16] and TNF-α [17, 18]. Additionally, resistance training for seven weeks led to a decrease in the expression of pro-inflammatory and profibrotic gene networks, signifying the local anti-inflammatory effects of exercise [19]. Moreover, there is substantial evidence indicating that a single bout of moderate to vigorous exercise induces increases in most major leukocyte subtypes within the circulation system [20–22].

However, despite the wealth of knowledge about the adverse effects of short sleep on the immune system, leading to systemic inflammation, and the well-established anti-inflammatory effects of exercise, most previous studies examining the mitigating effects of exercise on short sleep have predominantly focused on various other crucial health-related aspects, often overlooking inflammation. These areas encompass aging process [23], metabolic health [24], cognitive performance [25–27], cardiovascular function [28–30], insulin resistance [31], mental health [32–34], mitochondrial function [31, 35], and all-cause and cause-specific mortality risks [36].

This study aims to fill this knowledge gap by investigating the potential mitigating role of regular exercise in systemic inflammation for the short sleep population. Our primary aim is to explore the dose-response relationship between physical exercise and systemic inflammation, as assessed by the systemic immune inflammation index (SII), in individuals experiencing insufficient sleep. SII was selected as the principal indicator not only due to its rising prominence as a robust inflammation marker but also for its ability to predict various diseases, including myocardial infarction [37], coronary disease [38], hypertension [39], renal disease [40] and cancer [41].

The significance of this study lies in a comprehensive dose-effect relationship between exercise and SII in individuals with short sleep duration. The findings not only contribute to a deeper understanding of the intricate interplay between exercise, immune response, and short sleep but also offer practical recommendations for exercise regiments tailored to individuals facing the challenges of inadequate sleep.

## Methods

### Study design and participant’s inclusion

The present study drew upon the NHANES dataset, a pivotal component within a series of health-related programs executed by the Centers for Disease Control and Prevention’s (CDC) National Center for Health Statistics (NCHS) [42]. In an endeavor to capture comprehensive trends in the prevalence of select diseases and to delve into the intricate interplay linking diet, nutrition, and health, we amalgamated data from six release cycles: 2007–2008, 2009–2010, 2011–2012, 2013–2014, 2015–2016, and 2017–2018. This dataset comprised two distinct segments, each bearing its own distinctive attributes. Initially, eligible participants were administered a survey questionnaire, conducted in their homes. These questionnaires garnered a trove of person-level demographics, in addition to insights into the realms of health and nutrition. Subsequently, participants were invited to grace the mobile examination centers (MECs), outfitted to facilitate standardized health examinations with precision and accuracy. Research procedure of NHANES was approved by the Institutional Review Board (IRB) of the National Center for Health Statistics (NCHS), with written informed consent obtained.

**Fig. 1:**
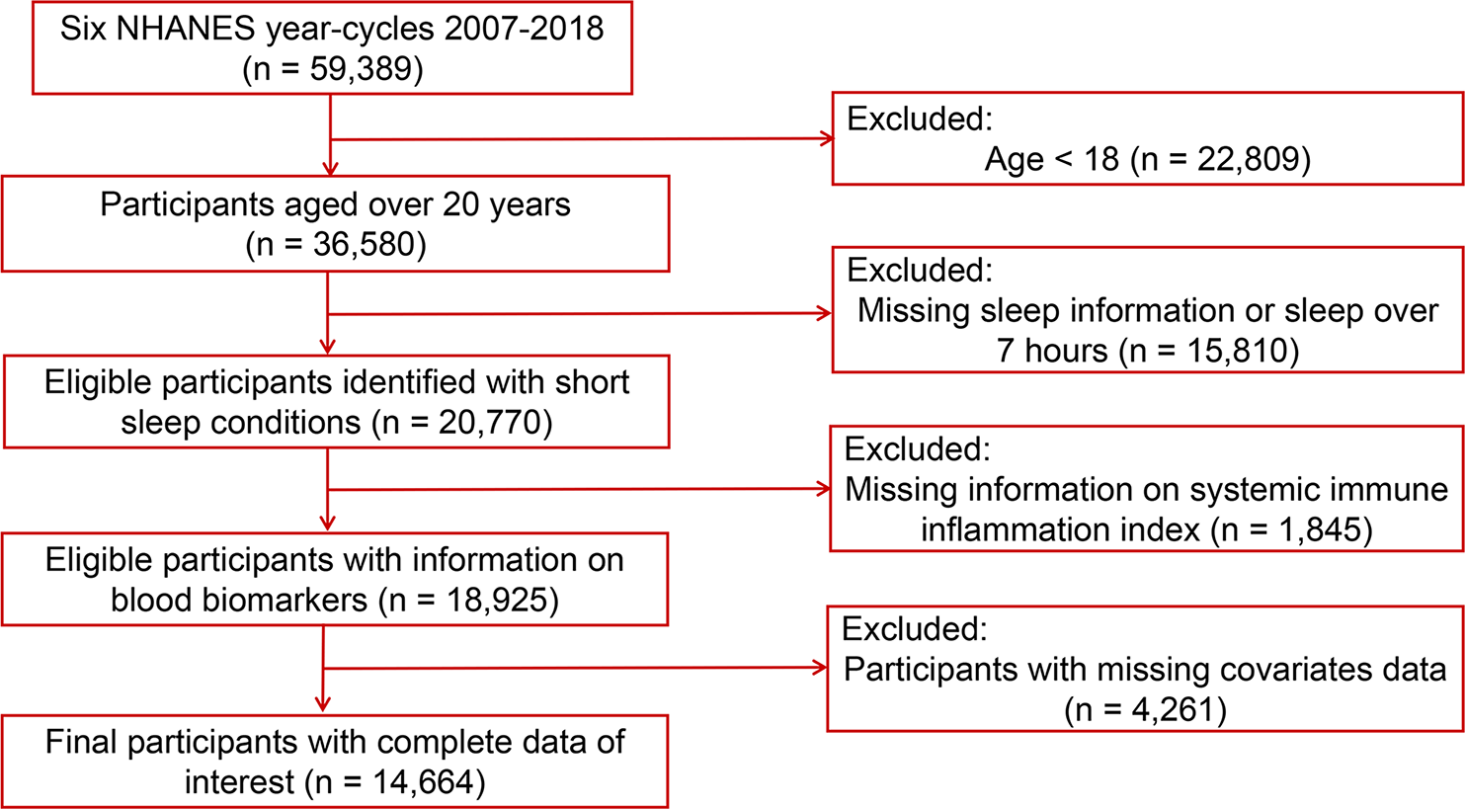
Flowchart of study population

Short sleep was identified as sleep no more than 7 h by sleep questionnaires [43, 44]. Throughout the data curation process, we scrutinized an initial 59,389 data records, employing rigorous inclusion and exclusion criteria. Specifically, 22,809 participants were excluded due to their tender age, as they had yet to attain the threshold of 18 years. Furthermore, an additional 15,810 participants were deemed ineligible for analysis. Their exclusion predicated upon either incomplete sleep questionnaire responses or an excess of 7 h of sleep. Additionally, 1,845 participants were excluded owing to the absence of biomarkers indispensable for the calculation of SII, and finally, 4,261 participants were removed from the analytical cohort on account of insufficient data pertaining to select covariates. Ultimately, our analysis focused on the remaining cohort of 14,664 adults. The graphical depiction illustrating the composition and progression of the study sample is shown in Fig. 1.

### Measurement of physical exercise level

Physical exercise (PE) was the exposure variable investigated in this study, encompassing a spectrum of leisure-time physical activities, such as sports, fitness endeavors, and various recreational pursuits. To gauge the frequency and patterns of PE, participants in NHANES were subjected to the Physical Activity Questionnaire (PAQ) [45]. Within this assessment, participants self-reported their activity patterns by recalling the nature, frequency (measured in exercise days per week), and duration (measured in exercise times per day) of their PE endeavors spanning the preceding 7-day window, with each engagement necessitating a minimum duration of 10 min. This assessment encompassed both moderate and vigorous intensity activities.

Defining the bounds of moderate and vigorous intensity PE activities proved pivotal, as it was relevant to recreational endeavors requiring varying degrees of physical effort. Referring to previous literature [27, 46], the metabolic equivalent of task (MET) was used as the tool to facilitate an evaluation of the physiological intensity associated with PE. Elucidating the calculation method of MET, it quantitatively gauged the energy expenditure incurred by a specific motion, with each MET corresponding to an oxygen consumption of 3.5 ml O^2^ kg^− 1^/min [45]. Within this framework, the recommended MET value for vigorous PE activities stood at 8.0, while that for moderate PE activities was set at 4.0. Mindful of the notion that a single-unit change in MET might not satisfactorily encapsulate the cumulative impact of a singular exercise event, we embarked upon an endeavor to introduce greater precision within our analytical framework. To this end, we devised an alternative unit of assessment, harnessing 100 * MET-min/week as a more comprehensive metric to portray the evolving trends within the realm of PE. By multiplying the number of days devoted to a particular activity by the average duration of that activity, and subsequently summing these products for each distinct activity, we could derive activity values. Ultimately, by dividing this cumulative value by 100, we can get the quantitative method to calculate the final PE volume reflected by 100 * MET-min/week. Given that the WHO recommends at least 150 min of moderate-intensity physical activity per week for healthy adults (equivalent to approximately 600 MET), we chose a threshold of 600 as the categorical variable for our analysis to explore the relationship between PE and SII in short sleep individuals who met and exceeded the recommended amount.

### Measurement of systemic immune inflammation index

Blood tests, specifically the complete blood count (CBC), were procured from the NHANES database, stemming from the endeavors of the NHANES Mobile Examination Center (MEC). Subsequently, the gathered blood samples underwent thorough analysis via automated hematology analyzing devices, with the Coulter DxH 800 analyzer serving as the instrument of choice. Within this analytical framework, crucial variables of interest, namely neutrophil count, lymphocyte count, and platelet count, were recorded. The calculation of the systemic immune inflammation index (SII), a metric capturing immune-inflammatory responses, hinged upon the integration of CBC values. To derive the SII, a formula was employed, as denoted by the following equation: platelet count multiplied by the neutrophil count, divided by the lymphocyte count [47, 48].

### Covariate measurement

Drawing upon the findings of several pertinent prior studies [49, 50], this investigation incorporated a comprehensive array of covariates deemed influential upon the obtained results. The covariates considered in this study encompassed age, sex, race, education level, marital status, poverty-to-income ratio (PIR), body mass index (BMI), smoking status, alcohol status, diabetes, cardiovascular diseases, and hypertension.

Age, was categorized into three quantiles: individuals below the age of 44, those aged 44 to 60, and individuals aged 60 and above. Sex, was dichotomized into male and female. The covariate of race encompassed four categories, including White, Black, Mexican, and other races. Education level, was delineated based on the attainment of educational milestones, specifically below high school level, high school completion, and attainment of college education or above. Marital status included never married, married / living with partner, and widowed / divorced. Poverty-to-income ratio (PIR), was calculated by dividing family income by the poverty guidelines specific to the survey year. This covariate was further classified into three categories, namely, PIR values below 1, PIR values ranging from 1 to 3, and PIR values equal to or exceeding 3. Body mass index (BMI), an integral component of health assessment, was discretized into three groups, reflecting BMI values below 25, BMI values ranging from 25 to 30, and BMI values equal to or exceeding 30. Furthermore, the covariate of smoking status assumed vital importance, adopting a tripartite classification encompassing never smokers, former smokers, and current smokers. Alcohol status, another pivotal covariate, was stratified into three distinctive groups, namely nondrinkers, individuals characterized by moderate alcohol use, and those exhibiting high levels of alcohol consumption. Details of the classification of alcohol use status can be found elsewhere [51].

Health conditions of paramount significance were also incorporated as covariates. Diabetes status was dichotomized as either present or absent. Similarly, cardiovascular diseases and hypertension were coded as dichotomous variables, designating their presence or absence within the studied population. The inclusion of these covariates reflects a comprehensive approach to account for their potential influence on the investigated relationships, fostering an enhanced understanding of the interplay between physical exercise and health outcomes.

### Statistical analyses

Statistical analysis was conducted utilizing R Software (version 4.2). To account for the intricacies of the complex survey design, encompassing factors such as unequal selection probabilities, appropriate adjustments were made by incorporating sampling weights during the data analysis process. Weighting processing and comprehensive analyses were performed on the final dataset to ensure the validity and representativeness of the results obtained. Detailed information regarding the survey procedures can be found elsewhere [52, 53].

Weighted means and standard errors were employed to present continuous variables, while weighted percentages were utilized for categorical variables. To examine the association between PE and the SII within complex survey samples, while adjusting for relevant covariates, a weighted generalized linear regression model was employed. Three distinct models were implemented: The Crude Model (unadjusted), Model 1 (adjusted for age, sex, and race/ethnicity), and Model 2 (further adjusted for body mass index, education, marital status, poverty status, smoking status, alcohol status, diabetes, cardiovascular diseases, and hypertension). Additionally, a two-piecewise linear regression model was developed to explore potential threshold effects and account for confounding factors. The determination of the threshold level for PE (expressed as 100 * MET-minutes/week) involved a recurrence method, entailing the identification of the inflection point within a predetermined interval, ultimately selecting the inflection point that yielded the maximum likelihood model. To compare the two-piecewise linear regression model with the one-line linear model, the log-likelihood ratio test was employed. Employing restricted cubic spline plots (RCS), we further detected potential nonlinear relationships between PE and the SII, adjusting for relevant covariates.

All statistical tests were two-sided, and a p-value below 0.05 was deemed statistically significant, signifying the presence of meaningful associations within the investigated framework.

## Results

The present study comprised a robust sample size, incorporating a total of 14,664 participants, reflecting a weighted population estimate of 102,074,864 individuals in the United States. Among the participants, 7,728 individuals (52.7%) were male, and 6,939 individuals (47.3%) were female. The distribution of participant characteristics, encompassing demographic and sociocultural dimensions, can be found in Table 1. Notably, 67.5% of the participants identified themselves as Non-Hispanic White, indicating a predominant racial composition within the cohort. Moreover, a substantial proportion of participants, accounting for 62.5% of the sample, had attained a college-level education or higher. The mean volume of physical exercise (PE) among the participants was quantified at 884 MET-minutes/week, reflecting engaging in 110.5 minutes’ vigorous intensity activities or 221 minutes’ moderate intensity activities per week. Furthermore, the mean score for the systemic immune inflammation index (SII), was recorded as 537.38 within our population.

**Table 1:** Demographic characteristics of study participants in NHANES 2007–2018

Variable	(%)*		Variable	(%/Mean)*
Age			Smoking status	
< 44	43.77		Never smoker	54.39
(44, 60)	33.89		Former smoker	24.59
≥ 60	22.34		Current smoker	21.02
Sex			Alcohol status	
Male	52.69		Nondrinker	23.24
Female	47.31		Moderate alcohol use	54.18
Race/ethnicity			High alcohol use	22.58
Non-hispanic White	67.48		Diabetes mellitus	
Non-hispanic Black	11.63		No	84.71
Mexican American	7.89		Yes	15.29
Other Race/ethnicity	13.00		Cardiovascular diseases	
Marital status			No	91.99
Never married	17.78		Yes	8.01
Married / living with partner	63.93		Hypertension	
Widowed / divorced	18.29		No	62.30
Education			Yes	37.70
Below high school	4.16		PE (as category variable, 100 * MET-minutes/week)	
High school	33.30		None	45.24
College or above	62.54		[1, 6)	16.47
Poverty income ratio			≥ 6	38.28
< 1	13.59		PE (100 * MET-minutes/week)	8.84 ± 0.22
(1,3)	35.13		Sleep duration (hours/day)	6.19 ± 0.01
≥ 3	51.28		SII (10^9^/L)	537.38 ± 4.55
BMI (kg/m^2^)			Platelets (10^9^/L)	243.7 ± 0.89
< 25	27.99		Neutrophils (10^9^/L)	4.32 ± 0.02
(25, 30)	33.40		Lymphocytes(10^9^/L)	2.14 ± 0.02
≥ 30	38.61			

Notes*Weighted percentage (%) for category variables and weighted Mean ± SE for continuous variables: NHANES, National Health and Nutrition Examination Survey; PE, physical exercise; BMI, body mass index; MET, metabolic equivalent of task

The relationship between PE and SII levels is elucidated in Table 2. Both continuous and categorized assessments of PE were undertaken to discern the nature and magnitude of this association. Notably, when PE was evaluated as a continuous variable, a consistent and statistically significant negative association emerged across all three weighted linear regression models. In the Crude Model, the regression coefficient (β) for PE exhibited a value of -1.261 (95% confidence interval [CI]: -1.600, -0.922), highlighting a robust and significant negative association (*p* < 0.001). Similarly, Model 1 yielded a β value of -1.005 (95% CI: -1.344, -0.666), further substantiating the presence of a significant negative association (*p* < 0.001). These findings remained consistent even after incorporating a multitude of covariates in Model 2, where the β value for PE stood at -0.470 (95% CI: -0.827, -0.112), with a p-value of 0.011.

**Table 2:** Associations between physical exercise and systemic immune inflammation index in the short sleep population

PE (100 * MET-minutes/week)		Crude model^a^			Model 1^b^			Model 2^c^	
		β (95% CI)	*P*-value		β (95% CI)	*P*-value		β (95% CI)	*P*-value
PE as continuous variable		-1.261(-1.600,-0.922)	< 0.001		-1.005(-1.344, -0.666)	< 0.001		-0.470(-0.827, -0.112)	0.011
PE as category variable									
None		Reference			Reference			Reference	
[1, 6)		-35.014(-55.279,-14.749)	< 0.001		-38.017(-58.132,-17.902)	< 0.001		-25.042(-46.400, -3.684)	0.022
≥ 6		-60.321(-74.000,-46.643)	< 0.001		-57.862(-71.665,-44.059)	< 0.001		-34.309(-50.153, -18.465)	< 0.001

*Notes*^a^Crude model, no covariates were adjusted. ^b^Model 1, age, sex, race/ethnicity were adjusted. ^c^Model 2, age, sex, race/ethnicity, body mass index, education, marital status, poverty status, smoking status, alcohol status, and chronic disease conditions were adjusted. PE, physical exercise; CI, confidence interval

The exploration of PE as a categorized variable further reinforced the consistent nature of these associations. Notably, in Model 2, after adjustment for a range of socio-demographic and health-related factors, the reference group comprised individuals who reported no engagement in PE. For those individuals involved in 1 to 600 MET-minutes/week of PE, a β value of -25.042 (95% CI: -46.400, -3.684) was observed, with a p-value of 0.022, indicative of significantly lower SII levels within this cohort. Furthermore, for individuals engaging in over 600 MET-minutes/week of PE, a robust negative association was evident, with a β value of -34.309 (95% CI: -50.153, -18.465), further corroborating the presence of a significant impact on SII levels (*p* < 0.001). These findings underscore the salient role of PE in mitigating systemic immune-inflammation in the short sleep population. Moreover, we conducted a subgroup analysis (Table S1). The findings, when stratified by various demographic factors, consistently revealed a notable correlation.

A log-likelihood ratio test (Table 3) was performed to compare the two non-segmented and segmented regression models and determine whether there existed a saturation effect of PE. The results of this analysis indicated statistical significance (*p* = 0.001), compelling us to explore the feasibility of a segmented regression model. Employing a two-piecewise linear regression model, we determined the inflection point to be situated at 2400 MET-minutes/week, demarcating a critical threshold within the examined relationship. Intriguingly, on the left of this inflection point, a significant association between PE and SII level was discerned. The regression coefficient (β) exhibited a value of -1.597 (95% CI: -2.374, -0.819), with a p-value below the threshold of statistical significance (< 0.001). This observation attests to a meaningful and negative relationship between PE and SII level within this segment of the data. Conversely, on the right of the inflection point, no discernible relationship between PE and SII level was observed. The regression coefficient (β) acquired a value of 0.396 (95% CI: -0.247, 1.038), alongside a corresponding p-value of 0.227.

**Table 3:** Saturation effect analysis of associations between physical exercise and systemic immune inflammation index in the short sleep population

	β (95% CI)	*P*-value
One - line linear regression model	-0.470 (-0.827, -0.112)	0.011
Two - piecewise linear regression model		
PE < 24 (100 * MET-minutes/week)	-1.597 (-2.374, -0.819)	< 0.001
PE ≥ 24 (100 * MET-minutes/week)	0.396 (-0.247, 1.038)	0.227
Log - likelihood ratio test		0.001

*Notes* Model 2 was used. Age, sex, race/ethnicity, body mass index, education, marital status, poverty status, smoking status, alcohol status, and chronic disease conditions were adjusted. PE, physical exercise; CI, confidence interval

**Fig. 2:**
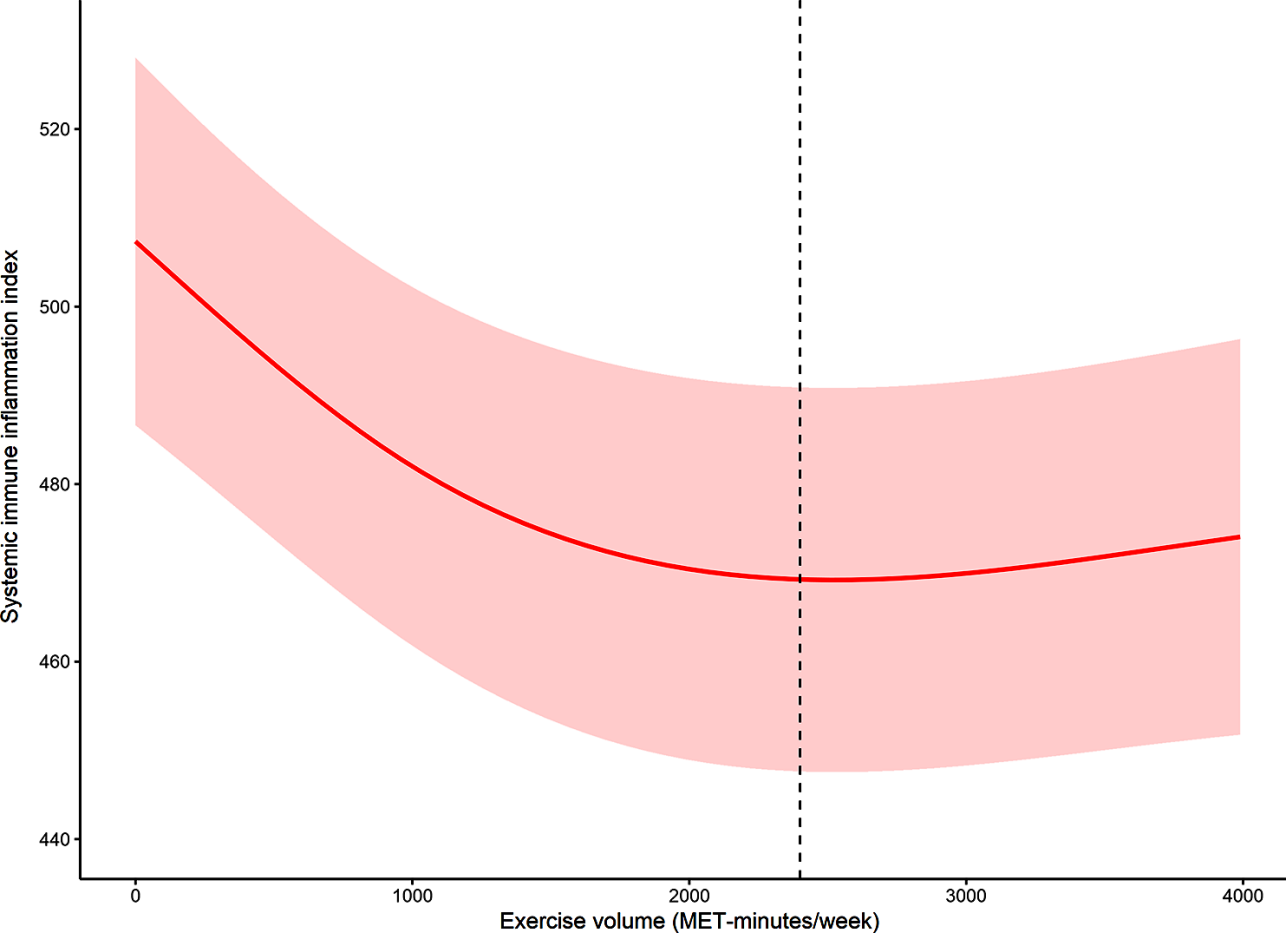
The dose-response relationship between physical exercise and systemic immune inflammation index in the short sleep population

Given the continuous nature of the PE variable, it was necessary to explore potential non-linear relationships within the analysis framework. The unit of PE was represented as MET-minutes/week within Fig. 2. After adjustment for relevant covariates such as age, sex, race/ethnicity, body mass index, education, marital status, poverty status, smoking status, alcohol status, and chronic disease conditions, the restricted cubic spline model revealed a discernible non-linear relationship between PE and SII level. In Fig. 2, the red solid line represents the β coefficient, thereby effectively capturing the magnitude and direction of the association. Furthermore, the dotted lines serve to delineate the point-wise 95% confidence intervals, contributing a comprehensive understanding of the uncertainty inherent in the estimated relationship.

## Discussions

Our investigation into the dose-effect relationship between exercise and systemic inflammation in short-sleep individuals revealed a significant inverse relationship between physical exercise and the SII in individuals with short sleep duration, which is consistent across all regression models. This noteworthy observation remained robust even after various covariates were adjusted.

The positive effects of physical activity were validated in this study. Comparing the changes to previous literature, a recent study observed that one MET increase in physical activity was associated with a decrease of 0.0005 SII [β (95% CI): − 0.0005 (− 0.0008, − 0.0002), *p* = 0.005] [54]. Moreover, there is evidence that SII mediated the relationship between sedentary behavior and sleep disturbance (mediation effect = 0.0003, *p* = 0.025) [55]. However, our comprehensive analyses, including the log-likelihood ratio test and subsequent two-piecewise linear regression modeling, not only support the presence of a saturation effect but also pinpoint a critical threshold at 2400 MET-minutes/week in the short sleep population. Below this inflection point, a significant and negative association between exercise and SII levels is observed, suggesting that more exercise within this range is beneficial to health by reducing systemic inflammation. Conversely, excessive exercise beyond this range was not significantly associated with lower SII, indicating the presence of a saturation effect, a novel and distinctive finding in our study. Vigorous intensity exercises have been reported to increase the risk of operatic infection of some diseases such as upper respiratory illness [56] and studies are showing that high-intensity exercise can promote pro-inflammation [57–59], indicating the adverse effect of exercise on the immune system when its intensity goes too high, which partially explain the presence of inflecting point we found.

While regular exercise is known to modulate inflammation through several pathways, including the reduction of pro-inflammatory cytokines and enhancement of anti-inflammatory response, the precise mechanism remains only partially understood [60]. According to previous studies analyzing the physiology and biology mechanism of the effect of exercise and short sleep on systemic inflammation, the key to the interaction between sleep, exercise, and inflammation may lie in the cytokines released during exercise and stress hormones increased by exercise and sleep, suggesting an intricate balance between exercise and inflammation, which may explain the does-effect relationship and duration effect between exercise and systemic inflammation in short-sleep individuals. First of all, the production and release, from skeletal muscle, of anti-inflammatory myokines can be increased by exercise, such as IL-6 mentioned before and IL-15 and irisin [61]. Secondly, the level of stress hormones can be increased by physical exercise, such as growth hormone, cortisol, adrenaline, and other hormones possessing immunomodulatory effects [62]. Even though there have been several studies linking increased inflammation with intense long exercise [63–65], which is consistent with the saturation effect of exercise on SII, more investigations are required to explain the reason behind the saturation effect of exercise on SII on individuals with short sleep duration.

In the broader context of exercise activity research, these results align with existing literature that documents a dose-response relationship between physical exercise and various health outcomes [66–69], including all-cause mortality, diabetes, breast cancer, ischemic heart disease, depression, cardiovascular health, and obesity. These studies typically emphasize the linear or near-linear benefits of increased activity. In contrast, the concept of a saturation point, as observed in our study, suggests a more complex interaction between exercise volume and health benefits, particularly in the context of inflammation response among those with insufficient sleep.

The strength of this study is the generalizability of our findings, which using data from a large, nationwide representative samples of NHANES. Our findings are consistent with previous research on the anti-inflammatory effects of exercise, but what distinguishes our study is our focus on the specific population of individuals suffering from short sleep. By delving into this underexplored area, we provide valuable insights into how exercise can be used as an effective non-pharmacological method to manage systemic inflammation and reduce health risks for short-sleep individuals.

However, it is important to acknowledge certain limitations of our study, including the reliance on self-reported exercise data and the potential for residual confounding factors. We also recognize the limitation of lacking causality analysis. In the future, collaboration with experts in population and exercise science will be crucial to delve into the specific molecular mechanisms of exercise’s anti-inflammatory properties and the background secrets of the saturation effect. Longitudinal studies will provide valuable insights into the long-term effects of physical exercise on inflammation in individuals facing short sleep.

## Conclusion

In conclusion, our study demonstrated the potential of exercise in mitigating systemic inflammation among short-sleep individuals. We have explored the dose-effect relationship, identified a saturation effect, and assumed underlying biological mechanisms. This suggested that performing no more than 2400 MET-minutes/week PE was associated with lower SII levels in the short sleep population, while more PE might not bring additional benefits. Despite study limitations, our findings provided valuable insights for managing inflammation in the context of short sleep. Further biological studies are warranted to establish causality and explore the physiological mechanism in this relationship.

### Electronic supplementary material

Below is the link to the electronic supplementary material.

Supplementary Material 1

## Data Availability

The data of original study are available in the NHANES repository. These data can be accessed using the following link: https://wwwn.cdc.gov/nchs/nhanes/Default.aspx. The datasets used and/or analyzed during the current study are available from the corresponding author on reasonable request.

## References

[1] Gallicchio L, Kalesan B (2009). Sleep duration and mortality: a systematic review and Meta-analysis. J Sleep Res.

[2] Cappuccio FP, D’Elia L, Strazzullo P, Miller MA (2010). Sleep duration and all-cause mortality: a systematic review and Meta-analysis of prospective studies. Sleep.

[3] Hirshkowitz M, Whiton K, Albert SM, Alessi C, Bruni O, DonCarlos L (2015). National Sleep Foundation’s Sleep Time duration recommendations: methodology and results Summary. Sleep Health.

[4] Shan Z, Ma H, Xie M, Yan P, Guo Y, Bao W (2015). Sleep duration and risk of type 2 diabetes: a Meta-analysis of prospective studies. Diabetes Care.

[5] Schmid SM, Hallschmid M, Schultes B (2015). The metabolic burden of sleep loss. Lancet Diabetes Endocrinol.

[6] Tobaldini E, Costantino G, Solbiati M, Cogliati C, Kara T, Nobili L et al. Sleep, Sleep Deprivation, Autonomic Nervous System and Cardiovascular Diseases. *Neurosci Biobehav Rev* (2017) 74(Pt B):321-9. Epub 2016/07/12. 10.1016/j.neubiorev.2016.07.004. 10.1016/j.neubiorev.2016.07.004 27397854

[7] Besedovsky L, Lange T, Haack M (2019). The Sleep-Immune Crosstalk in Health and Disease. Physiol Rev.

[8] Vgontzas AN, Papanicolaou DA, Bixler EO, Lotsikas A, Zachman K, Kales A (1999). Circadian Interleukin-6 secretion and quantity and depth of Sleep. J Clin Endocrinol Metab.

[9] Wright KP, Drake AL, Frey DJ, Fleshner M, Desouza CA, Gronfier C (2015). Influence of Sleep Deprivation and Circadian Misalignment on Cortisol, inflammatory markers, and Cytokine Balance. Brain Behav Immun.

[10] Kelly P, Kahlmeier S, Gotschi T, Orsini N, Richards J, Roberts N et al. Systematic Review and Meta-Analysis of Reduction in All-Cause Mortality from Walking and Cycling and Shape of Dose Response Relationship. *Int J Behav Nutr Phys Act* (2014) 11:132. Epub 2014/10/26. 10.1186/s12966-014-0132-x. 10.1186/s12966-014-0132-x PMC4262114 25344355

[11] You Y, Chen Y, Zhang Y, Zhang Q, Yu Y, Cao Q (2023). Mitigation Role of Physical Exercise participation in the relationship between blood cadmium and sleep disturbance: a cross-sectional study. BMC Public Health.

[12] You Y, Li W, Liu J, Li X, Fu Y, Ma X (2021). Bibliometric Review To Explore Emerging High-Intensity Interval Training in Health Promotion: a New Century Picture. Front Public Health.

[13] Garber CE, Blissmer B, Deschenes MR, Franklin BA, Lamonte MJ, Lee IM (2011). American College of Sports Medicine Position Stand. Quantity and quality of Exercise for developing and maintaining Cardiorespiratory, Musculoskeletal, and Neuromotor Fitness in apparently healthy adults: Guidance for Prescribing Exercise. Med Sci Sports Exerc.

[14] Fedewa MV, Hathaway ED, Ward-Ritacco CL (2017). Effect of Exercise Training on C reactive protein: a systematic review and Meta-analysis of randomised and non-randomised controlled trials. Br J Sports Med.

[15] Svensson M, Lexell J, Deierborg T (2015). Effects of Physical Exercise on Neuroinflammation, Neuroplasticity, Neurodegeneration, and Behavior: what we can learn from animal models in clinical settings. Neurorehabil Neural Repair.

[16] Bobinski F, Ferreira TAA, Cordova MM, Dombrowski PA, da Cunha C, Santo C (2015). Role of Brainstem Serotonin in Analgesia produced by low-intensity Exercise on Neuropathic Pain after sciatic nerve Injury in mice. Pain.

[17] Hong S, Dimitrov S, Pruitt C, Shaikh F, Beg N. Benefit of Physical Fitness against Inflammation in Obesity: Role of Beta Adrenergic Receptors. *Brain Behav Immun* (2014) 39:113 – 20. Epub 2013/12/21. 10.1016/j.bbi.2013.12.009. 10.1016/j.bbi.2013.12.009 PMC4059789 24355098

[18] Petersen AM, Pedersen BK (2005). The anti-inflammatory effect of Exercise. J Appl Physiol (1985).

[19] Nader GA, Dastmalchi M, Alexanderson H, Grundtman C, Gernapudi R, Esbjornsson M (2010). A longitudinal, Integrated, Clinical, histological and Mrna Profiling Study of Resistance Exercise in Myositis. Mol Med.

[20] Nieman DC, Wentz LM (2019). The compelling link between physical activity and the body’s Defense System. J Sport Health Sci.

[21] Simpson RJ, Kunz H, Agha N, Graff R. Exercise and the Regulation of Immune Functions. *Prog Mol Biol Transl Sci* (2015) 135:355 – 80. Epub 2015/10/20. 10.1016/bs.pmbts.2015.08.001. 10.1016/bs.pmbts.2015.08.001 26477922

[22] Simpson RJ, Bigley AB, Agha N, Hanley PJ, Bollard CM (2017). Mobilizing Immune cells with Exercise for Cancer Immunotherapy. Exerc Sport Sci Rev.

[23] You Y, Liu J, Li X, Wang P, Liu R, Ma X (2024). Relationship between accelerometer-measured sleep duration and Stroop Performance: a functional near-infrared spectroscopy study among young adults. PeerJ.

[24] Monico-Neto M, Antunes HK, Lee KS, Phillips SM, Giampa SQ, Souza Hde S (2015). Resistance Training minimizes Catabolic effects Induced by Sleep Deprivation in rats. Appl Physiol Nutr Metab.

[25] Sauvet F, Arnal PJ, Tardo-Dino PE, Drogou C, Van Beers P, Erblang M (2020). Beneficial effects of Exercise Training on cognitive performances during total sleep deprivation in healthy subjects. Sleep Med.

[26] You Y, Liu J, Wang D, Fu Y, Liu R, Ma X. Cognitive Performance in Short Sleep Young Adults with Different Physical Activity Levels: A Cross-Sectional Fnirs Study. *Brain Sci* (2023) 13(2). Epub 2023/02/26. 10.3390/brainsci13020171. 10.3390/brainsci13020171 PMC9954673 36831714

[27] You Y, Mo L, Tong J, Chen X, You Y (2024). The role of Education Attainment on 24-Hour Movement Behavior in emerging adults: evidence from a Population-based study. Front Public Health.

[28] Stockelman KA, Bain AR, Dow CA, Diehl KJ, Greiner JJ, Stauffer BL (2021). Regular aerobic Exercise counteracts Endothelial Vasomotor Dysfunction Associated with Insufficient Sleep. Am J Physiol Heart Circ Physiol.

[29] Kizilkilic SE, Falter M, Dendale P (2023). The power of Movement: how physical activity can mitigate the risks of inadequate sleep. Eur J Prev Cardiol.

[30] Chen CW, Kuo TBJ, Hsu PC, Li JY, Kuo KL, Yang CCH (2022). Roles of sleep-related Cardiovascular autonomic functions in Voluntary-Exercise-Induced alleviation of hypertension in spontaneously hypertensive rats. Hypertens Res.

[31] Saner, Bishop, Bartlett (2018). Is Exercise a viable therapeutic intervention to mitigate mitochondrial dysfunction and insulin Resistance Induced by Sleep loss?. Sleep Med Rev.

[32] Larsen P, Marino FE, Guelfi K, Duffield R, Skein M (2021). A preliminary investigation of the effects of Short-Duration, vigorous Exercise following Sleep Restriction, Fragmentation and Extension on Appetite and Mood in Inactive, Middle-aged men. J Sleep Res.

[33] Tai F, Wang C, Deng X, Li R, Guo Z, Quan H (2020). Treadmill Exercise ameliorates chronic Rem Sleep Deprivation-Induced anxiety-like Behavior and Cognitive Impairment in C57bl/6j mice. Brain Res Bull.

[34] You Y, Wang R, Li J, Cao F, Zhang Y, Ma X. The Role of Dietary Intake of Live Microbes in the Association between Leisure-Time Physical Activity and Depressive Symptoms: A Population-Based Study. *Appl Physiol Nutr Metab* (2024). Epub 2024/04/03. 10.1139/apnm-2023-0550. 10.1139/apnm-2023-0550 38569203

[35] Saner NJ, Lee MJ, Kuang J, Pitchford NW, Roach GD, Garnham A (2021). Exercise mitigates sleep-loss-Induced changes in glucose tolerance, mitochondrial function, sarcoplasmic protein synthesis, and diurnal rhythms. Mol Metab.

[36] Liang YY, Feng H, Chen Y, Jin X, Xue H, Zhou M (2023). Joint Association of Physical Activity and Sleep Duration with risk of all-cause and cause-specific mortality: a Population-based Cohort Study using Accelerometry. Eur J Prev Cardiol.

[37] Zhu Y, He H, Qiu H, Shen G, Wang Z, Li W (2023). Prognostic value of systemic Immune-inflammation index and Nt-Probnp in patients with Acute St-Elevation myocardial infarction. Clin Interv Aging.

[38] Candemir M, Kiziltunc E, Nurkoc S, Sahinarslan A (2021). Relationship between systemic Immune-inflammation index (Sii) and the severity of stable coronary artery disease. Angiology.

[39] Karayigit O, Nurkoc SG, Celik MC (2023). Systemic Immune-inflammation index (Sii) May be an effective Indicator in Predicting the Left ventricular hypertrophy for patients diagnosed with hypertension. J Hum Hypertens.

[40] Chen JB, Tang R, Zhong Y, Zhou YO, Zuo X, Luo H (2021). Systemic Immune-inflammation index predicts a reduced risk of end-stage renal disease in Chinese patients with myeloperoxidase-anti-neutrophil cytoplasmic antibody-Associated Vasculitis: a retrospective observational study. Exp Ther Med.

[41] Li M, Li Z, Wang Z, Yue C, Hu W, Lu H (2022). Prognostic value of systemic Immune-inflammation index in patients with pancreatic Cancer: a Meta-analysis. Clin Exp Med.

[42] Cdc/National Center for Health Statistics Nhanes. Overview [cited 2023 4 October]. https://www.cdc.gov/nchs/nhanes/index.htm.

[43] You Y, Chen Y, Chen X, Wei M, Yin J, Zhang Q (2023). Threshold effects of the relationship between Physical Exercise and cognitive function in the short-sleep elder Population. Front Aging Neurosci.

[44] You Y, Li J, Zhang Y, Li X, Li X, Ma X (2024). Exploring the potential relationship between short sleep risks and cognitive function from the perspective of inflammatory biomarkers and Cellular pathways: insights from Population-based and mice studies. CNS Neurosci Ther.

[45] Hallal PC, Andersen LB, Bull FC, Guthold R, Haskell W, Ekelund U (2012). Global physical activity levels: Surveillance Progress, Pitfalls, and prospects. Lancet.

[46] Li Y, Tong WD, Qian Y (2021). Effect of physical activity on the Association between Dietary Fiber and Constipation: evidence from the National Health and Nutrition Examination Survey 2005–2010. J Neurogastroenterol Motil.

[47] Hu B, Yang XR, Xu Y, Sun YF, Sun C, Guo W (2014). Systemic Immune-inflammation index predicts prognosis of patients after curative resection for Hepatocellular Carcinoma. Clin Cancer Res.

[48] You Y, Chen Y, Wei M, Tang M, Lu Y, Zhang Q, et al. Mediation role of recreational physical activity in the relationship between the Dietary Intake of Live microbes and the systemic Immune-inflammation index: a real-world cross-sectional study. Nutrients. 2024;16(6). 10.3390/nu16060777. Epub 2024/03/28. 10.3390/nu16060777 PMC10974920 38542688

[49] Yin J, Gong R, Zhang M, Ding L, Shen T, Cai Y (2023). Associations between Sleep Disturbance, inflammatory markers and depressive symptoms: mediation analyses in a large Nhanes Community Sample. Prog Neuropsychopharmacol Biol Psychiatry.

[50] You Y, Chen Y, Zhang Q, Yan N, Ning Y, Cao Q (2023). Muscle Quality Index is Associated with trouble sleeping: a cross-sectional Population based study. BMC Public Health.

[51] Taylor AL, Denniston MM, Klevens RM, McKnight-Eily LR, Jiles RB (2016). Association of Hepatitis C Virus with Alcohol Use among U.S. adults: Nhanes 2003–2010. Am J Prev Med.

[52] Mirel LB, Mohadjer LK, Dohrmann SM, Clark J, Burt VL, Johnson CL (2013). National Health and Nutrition Examination Survey: estimation procedures, 2007–2010. Vital Health Stat.

[53] Chen TC, Parker JD, Clark J, Shin HC, Rammon JR, Burt VL (2018). National Health and Nutrition Examination Survey: estimation procedures, 2011–2014. Vital Health Stat.

[54] Guo L, Huang Y, He J, Li D, Li W, Xiao H (2024). Associations of Lifestyle characteristics with circulating Immune markers in the General Population based on Nhanes 1999 to 2014. Sci Rep.

[55] You Y, Chen Y, Fang W, Li X, Wang R, Liu J (2022). The Association between Sedentary Behavior, Exercise, and Sleep Disturbance: a mediation analysis of inflammatory biomarkers. Front Immunol.

[56] Kakanis MW, Peake J, Brenu EW, Simmonds M, Gray B, Hooper SL (2010). The Open window of susceptibility to infection after Acute Exercise in Healthy Young Male Elite athletes. Exerc Immunol Rev.

[57] Pinho RA, Silva LA, Pinho CA, Scheffer DL, Souza CT, Benetti M (2010). Oxidative stress and inflammatory parameters after an Ironman race. Clin J Sport Med.

[58] de Lucas RD, Caputo F, Mendes de Souza K, Sigwalt AR, Ghisoni K, Lock Silveira PC (2014). Increased platelet oxidative metabolism, blood oxidative stress and neopterin levels after Ultra-endurance Exercise. J Sports Sci.

[59] You Y (2024). Accelerometer-measured physical activity and sedentary Behaviour are Associated with C-Reactive protein in us adults who get Insufficient Sleep: a threshold and Isotemporal Substitution Effect Analysis. J Sports Sci.

[60] Scheffer DDL, Latini A (2020). Exercise-Induced Immune System Response: anti-inflammatory status on Peripheral and Central organs. Biochim Biophys Acta Mol Basis Dis.

[61] Pedersen BK (2017). Anti-inflammatory effects of Exercise: Role in Diabetes and Cardiovascular Disease. Eur J Clin Invest.

[62] Gleeson M, Bishop NC, Stensel DJ, Lindley MR, Mastana SS, Nimmo MA (2011). The anti-inflammatory effects of Exercise: mechanisms and implications for the Prevention and Treatment of Disease. Nat Rev Immunol.

[63] Bonsignore MR, Morici G, Riccobono L, Insalaco G, Bonanno A, Profita M (2001). Airway Inflammation in nonasthmatic amateur runners. Am J Physiol Lung Cell Mol Physiol.

[64] Degerstrom J, Osterud B (2006). Increased inflammatory response of blood cells to repeated Bout of endurance Exercise. Med Sci Sports Exerc.

[65] Bernecker C, Scherr J, Schinner S, Braun S, Scherbaum WA, Halle M (2013). Evidence for an Exercise Induced increase of tnf-alpha and Il-6 in Marathon runners. Scand J Med Sci Sports.

[66] You Y, Wei M, Chen Y, Fu Y, Ablitip A, Liu J (2023). The Association between recreational physical activity and depression in the short Sleep Population: a cross-sectional study. Front Neurosci.

[67] Geidl W, Schlesinger S, Mino E, Miranda L, Pfeifer K (2020). Dose-response relationship between physical activity and mortality in adults with noncommunicable diseases: a systematic review and Meta-analysis of prospective observational studies. Int J Behav Nutr Phys Act.

[68] Recchia F, Leung CK, Yu AP, Leung W, Yu DJ, Fong DY (2023). Dose-response effects of Exercise and caloric restriction on visceral adiposity in overweight and obese adults: a systematic review and Meta-analysis of Randomised controlled trials. Br J Sports Med.

[69] Pischon T, Hankinson SE, Hotamisligil GS, Rifai N, Rimm EB (2003). Leisure-time physical activity and reduced plasma levels of obesity-related inflammatory markers. Obes Res.

