# Supplementary material for: Saturation effects of the relationship between physical exercise and systemic immune inflammation index in the short-sleep population: a cross-sectional study
Source: BMC Public Health. 2024 Jul 17;24:1920. doi: 10.1186/s12889-024-19432-7 (PMC11256404; doi:10.1186/s12889-024-19432-7)
Supplement: Supplementary file 1 — Supplementary Material 1 [file 12889_2024_19432_MOESM1_ESM.docx]

Table S1. Stratified results for the association between physical exercise and systemic immune inflammation index in the short sleep population.

|  | None | Less than 600 MET-minutes/week | *P-value* | More than 600 MET-minutes/week | *P-value* | *P for trend* | *P for interaction* |
| --- | --- | --- | --- | --- | --- | --- | --- |
| Sex |  |  |  |  |  |  | 0.867 |
| Male | Reference | -35.895(-67.082, -4.708) | 0.025 | -61.87(-80.671,-43.069) | <0.001 | <0.001 |  |
| Female | Reference | -32.064(-55.804, -8.324) | 0.009 | -54.126(-75.535,-32.718) | <0.001 | <0.001 |  |
| Age |  |  |  |  |  |  | 0.527 |
| < 44 | Reference | -11.347(-41.637, 18.944) | 0.459 | -53.982(-72.042,-35.923) | <0.001 | <0.001 |  |
| [44, 60) | Reference | -46.509(-82.568,-10.451) | 0.012 | -63.044(-89.328,-36.760) | <0.001 | <0.001 |  |
| ≥ 60 | Reference | -43.467(-89.964, 3.030) | 0.067 | -57.952(-89.660,-26.243) | <0.001 | <0.001 |  |
| Race/ethnicity |  |  |  |  |  |  | 0.052 |
| Non-hispanic White | Reference | -43.027(-70.105,-15.949) | 0.002 | -79.298(-98.747,-59.849) | <0.001 | <0.001 |  |
| Non-hispanic Black | Reference | -29.389(-61.721, 2.943) | 0.074 | -41.648(-71.752,-11.545) | 0.007 | 0.006 |  |
| Mexican American | Reference | -34.672(-82.053,12.709) | 0.149 | -33.605(-73.175, 5.965) | 0.095 | 0.084 |  |
| Other Race/ethnicity | Reference | -20.219(-63.952,23.515) | 0.361 | -31.97(-65.827, 1.887) | 0.064 | 0.065 |  |
| Marital status |  |  |  |  |  |  | 0.455 |
| Never married | Reference | -23.306(-61.137, 14.524) | 0.224 | -42.707(-73.648,-11.765) | 0.007 | 0.008 |  |
| Married/living with partner | Reference | -40.812(-68.267,-13.358) | 0.004 | -68.373(-85.145,-51.601) | <0.001 | <0.001 |  |
| Widowed/ divorced | Reference | -10.717(-53.048,31.615) | 0.616 | -46.125(-85.558,-6.693) | 0.022 | 0.028 |  |
| Poverty income ratio |  |  |  |  |  |  | 0.375 |
| < 1 | Reference | -15.987( -53.088, 21.113) | 0.394 | -84.164(-113.317,-55.011) | <0.001 | <0.001 |  |
| [1,3) | Reference | -36.913(-74.072, 0.246) | 0.052 | -49.023(-72.949,-25.097) | <0.001 | <0.001 |  |
| ≥ 3 | Reference | -32.858(-60.729, -4.986) | 0.021 | -63.564(-83.786,-43.342) | <0.001 | <0.001 |  |
| Education |  |  |  |  |  |  | 0.723 |
| Below high school | Reference | -74.488(-128.736,-20.240) | 0.008 | -62.757(-107.639,-17.875) | 0.007 | 0.002 |  |
| High school | Reference | -40.317(-75.212, -5.421) | 0.024 | -56.829(-83.121,-30.537) | <0.001 | <0.001 |  |
| College or above | Reference | -28.192(-54.797, -1.587) | 0.038 | -62.265(-81.053,-43.477) | <0.001 | <0.001 |  |
| BMI(kg/m^2^) |  |  |  |  |  |  | 0.894 |
| < 25 | Reference | -48.296(-92.146, -4.447) | 0.031 | -61.458(-91.754,-31.163) | <0.001 | <0.001 |  |
| [25, 30) | Reference | -26.789(-62.903, 9.324) | 0.144 | -57.647(-79.757,-35.537) | <0.001 | <0.001 |  |
| ≥ 30 | Reference | -24.249(-51.275, 2.778) | 0.078 | -57.157(-83.750,-30.565) | <0.001 | <0.001 |  |
| Smoking status |  |  |  |  |  |  | 0.878 |
| Never smoker | Reference | -20.708(-43.507, 2.092) | 0.075 | -52.025(-70.351,-33.699) | <0.001 | <0.001 |  |
| Former smoker | Reference | -44.504(-86.043, -2.964) | 0.036 | -64.493(-97.338,-31.648) | <0.001 | <0.001 |  |
| Current smoker | Reference | -31.667(-84.701, 21.367) | 0.239 | -59.473(-91.017,-27.929) | <0.001 | <0.001 |  |
| Alcohol status |  |  |  |  |  |  | 0.393 |
| Nondrinker | Reference | -27.771( -78.769, 23.227) | 0.282 | -74.606(-107.446,-41.767) | <0.001 | <0.001 |  |
| Moderate alcohol use | Reference | -36.876(-64.309, -9.443) | 0.009 | -63.529(-81.445,-45.612) | <0.001 | <0.001 |  |
| High alcohol use | Reference | -14.645(-57.406,28.115) | 0.498 | -32.466(-60.905,-4.026) | 0.026 | 0.027 |  |
| Cardiovascular diseases |  |  |  |  |  |  | 0.028 |
| No | Reference | -40.906(-62.057,-19.754) | <0.001 | -62.781(-76.239,-49.323) | <0.001 | <0.001 |  |
| Yes | Reference | 80.124(-20.140,180.388) | 0.116 | -33.023(-87.964, 21.917) | 0.236 | 0.552 |  |
| Hypertension |  |  |  |  |  |  | 0.747 |
| No | Reference | -31.458(-65.221, 2.305) | 0.067 | -64.509(-90.036,-38.982) | <0.001 | <0.001 |  |
| Yes | Reference | -29.7(-55.697, -3.703) | 0.026 | -52.93(-68.282,-37.578) | <0.001 | <0.001 |  |
| Diabetes mellitus |  |  |  |  |  |  | 0.488 |
| No | Reference | -33.419(-59.243, -7.594) | 0.012 | -54.576(-70.135,-39.017) | <0.001 | <0.001 |  |
| Yes | Reference | -1.057(-50.935,48.821) | 0.967 | -31.823(-88.982,25.336) | 0.272 | 0.294 |  |
